# Release of Heavy Metals from Plastic Statuettes Used as Souvenirs and/or Toys Handled by Children

**DOI:** 10.3390/ijerph19010236

**Published:** 2021-12-26

**Authors:** Pietro Pandolfi, Maria Pia Sammartino, Giovanni Visco, Pasquale Avino, Virgilio Stillittano

**Affiliations:** 1 Department of Biomedicine and Prevention, University of Rome Tor Vergata, via Bardanzellu 8, I-00155 Rome, Italy; ppfpcd@tin.it; 2 Department of Chemistry, University of Rome “La Sapienza”, p.le Aldo Moro 5, I-00185 Rome, Italy; mariapia.sammartino@uniroma1.it (M.P.S.); giovanni.visco@uniroma1.it (G.V.); 3 Department of Agricultural, Environmental and Food Sciences (DiAAA), University of Molise, via De Sanctis, I-86100 Campobasso, Italy; 4 Istituto Zooprofilattico Sperimentale del Lazio e della Toscana “M. Aleandri”, via Appia Nuova 1411, I-00178 Roma, Italy; v.stillittano-esterno@sanita.it

**Keywords:** statuettes, heavy metals, leaching test, ICP-MS, cluster analysis, principal component analysis, human health, toxicity, children

## Abstract

Different plastic toys are on sale in the Italian market: they are sold as souvenirs and/or as toys. Such statuettes, called Gongoli, represent a famous character (a soccer player, a politician, the Pope, etc.). In particular, these products are widely sold, but the material composition is not sufficiently defined. Further, the effect of the release of dangerous compounds on human health is not sufficiently documented. Following this hypothesis, a study on eight different statuettes was carried out both for evaluating the possible presence of heavy metals and for evidencing their release from these objects. Preliminary analysis by means of EDS spectroscopy highlighted the percentage chemical composition of different products, especially the presence of total Cr and Ni. Release tests evidenced the release of Cr, Cu, Ni, and Pb: Pb reached 74 mg kg^−1^, which is an interesting value even if it is lower than reported in the legislation. This study should be considered preliminary due to its limitations, such as the number of items investigated and the large variability found for some elements, but it highlights a serious problem related to the classification of these products which are marketed as souvenirs but manipulated by children.

## 1. Introduction

Several studies have focused on the determination of heavy metal contamination in toys [1,2,3]. For example, Weidenhamer and co-authors [4,5] presented interesting findings in this field: they studied the release of some metals, particularly lead, in low-cost objects used by children. Although this topic has been sufficiently studied and investigated, millions of toys that are dangerous to the safety and health of consumers, and especially children [3,6,7], are seized every day by competent authorities around the world. For example, one of the main causes of this danger could be due to colorful toy cars and dolls: their plastics contain phthalates, some of which are shown to be carcinogenic or show properties which may be hazardous to human health [8,9]. In any case, the main problem concerns the content of heavy metals in the objects handled by children and adults. Contamination risk is very high, especially for children as a very sensitive subpopulation [10,11,12].

In recent years, several statuettes, commercially called “Gongoli”, have appeared in the market. These objects are used as souvenirs, but they could be considered as toys as they are especially used by children. The profiles of such figures are very different, e.g., a gladiator, or a football player. The statuettes have different colors and are made of a material very similar to that commonly referred to as “polyresin”, a resin compound generally used for statues, figurines, decorations, and furniture. This sturdy material can be molded, allowing for a great level of detail with a consistent degree of texture. Additives can be incorporated into the compound to improve some characteristics (for example, to increase the strength of the material, to reduce its weight, to add heat stability, and decorative effects). This material is also compatible with a wide range of different finishes, including paints and metallic finishes. It is a stone-based material, easy to carve, absorbs paint well, and looks like porcelain and ceramic. All these concerns open important safety and health issues due to the implications related to the handling of such objects and the possible release of chemicals (e.g., heavy metals, colored pigments, and organic compounds from varnishes and paints) [13]. This paper focuses the attention on the determination of heavy metals (and some organic substances) that may be present in such figurines. There are some papers in the literature on the release of heavy metals from plastic toys, but only a few articles are related to these types of objects which are widely marketed all over the world [14]. The methodologic approach was based on the preliminary chemical characterization of different figures using spectroscopic techniques (X-ray and EDS spectroscopy) followed by both a thorough investigation of the metal content and release tests at different times (24 h, 7 days, and 15 days). Finally, a chemometric study made it possible to identify which metals were mainly responsible for exposure.

## 2. Materials and Methods

### 2.1. Statuettes Investigated

**Figure 1:**
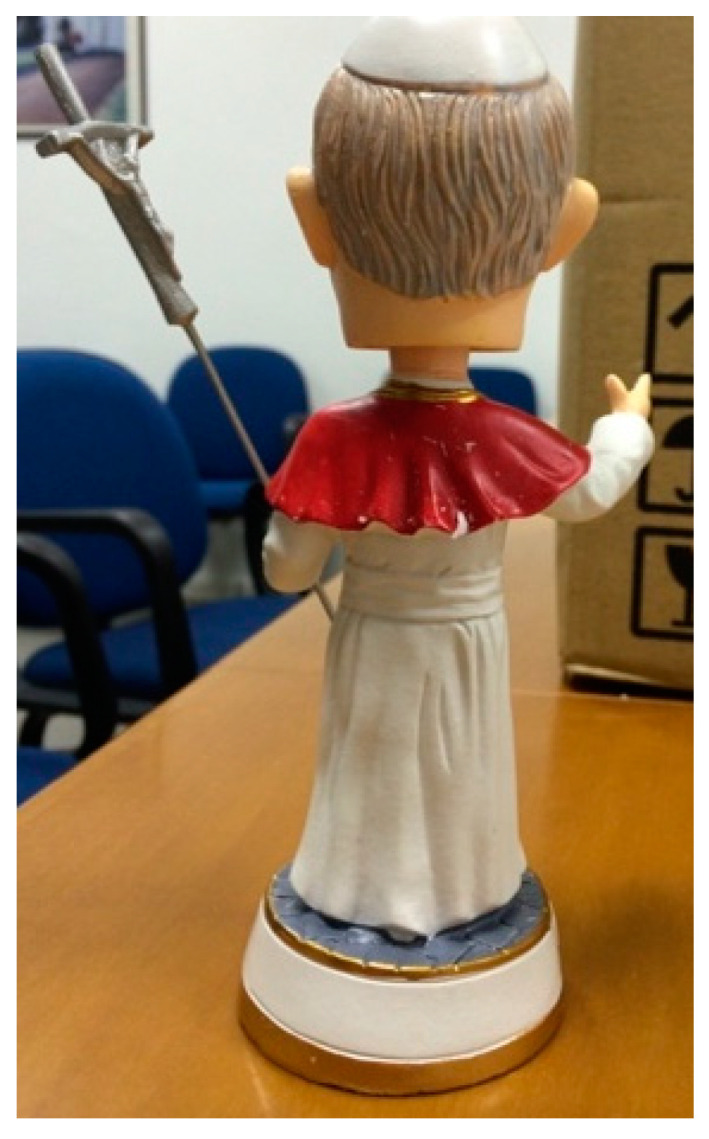
Example of a statuette investigated in this study.

The statuettes had different shapes and were about 15–20 cm in size, with different characters depicted and different colors. Eight different statuettes were analyzed in terms of heavy metal content (i.e., Pope with red dress, Pope with white dress, gladiator with black crest, gladiator with red crest, gladiator with laurel crown, Santa Klaus, centurion, footballer); the same statuettes with the addition of another 2 (i.e., gladiator with *gladius* and Pope with red cross and *zucchetto*) were involved in the release tests. Figure 1 shows an example of a sample investigated. The statuettes are available in the Italian market; they can be bought in any souvenir shop, but they are manufactured in China, as reported on the label. They were acquired in different places, but they were all from the same provenance/origin (same bar code) to avoid differences in the sampling.

### 2.2. Analytical Investigation

Preliminarily, for a better chemical investigation, measurements based on X-ray diffraction and energy-dispersive spectrometry (EDS) techniques were performed. The decision to use these analytical methods was determined by the need to deepen the study of the elemental and structural composition of these souvenirs/toys before carrying out a leaching study. For the X-ray analysis, it was decided to cut the figurines in question and take an internal section (of 1 mm). At the X-ray examination, the situation was complex: in fact, the spectrum area indicated that each sample was different from one another and that the material of which the figures were made was not a polymer. Following this preliminary information, a scanning electron microscope (SEM, mod. AURIGA, Carl Zeiss Microscopy GmbH, Jena, Germany) equipped with an energy-dispersive spectrometer for X-ray microanalysis (XEDS, model QUANTAX, Bruker Italia S.r.l., Milan, Italy) was used (acquisitions under high vacuum, 10^−6^ hPa at 20 keV accelerating voltage), whereas diffractometry was carried out with measurements at different angles of incidence, with an exposure of 12 h. The analysis showed the presence of several peaks which confirmed, at 99.99%, that the material in question was not a polymer with a well-defined composition. This hypothesis was subsequently confirmed by the analysis of 3 other samples which, after the analysis, showed totally different peaks. After, some heavy metals and organic compounds were determined. Finally, the focus was on the leaching tests at different times. These tests were carried out at 24 h, 7 days, and 15 days according to the CNR IRSA method [15]. Each statuette was put in contact with simulated artificial sweat solution (100 mL) prepared according to the Deutsches Institut für Normung (DIN) procedure [16]: sodium chloride (NaCl) 5.0 g L^−1^, urea 1.0 g L^−1^, lactic acid (>88%) 1.0 g L^- 1^, adjusted to pH 6.5 ± 0.1 with a solution of 1% ammonium hydroxide. After, analyses for the determination of metals were carried out with the previously reported time sequence. The metals were analyzed according to the official method UNI EN ISO 15587-1:2002 Annex A [17] implemented by UNI EN ISO 17294-2:2005 [18]. Such a guideline describes, in detail, a method for the determination of 62 elements by means of ICP-MS spectrometry. Briefly, element concentrations were determined by means of ICP-MS (^45^Sc and ^232^Th as internal standards to control the nebulizer efficiency) (mod. 7850, Agilent, Santa Clara, CA, USA), whereas mercury was analyzed using an atomic fluorescence spectrometer (AFS 8220-AS 06, FullTech Instruments, Rome, Italy). For measurements, primary standards, as single-element solutions at a concentration of 1000 μg mL^−1^, were used (Carlo Erba, Milan, Italy) as well as a multi-element standard solution (multi-element standard XIII, Merck KGaA, Darmstadt, Germany). The intra-day and inter-day precision and accuracy, <6% and <9%, respectively, calculated as relative standard deviation (RSD), were estimated by analyzing six replicates at four different concentration levels on the same day and on three consecutive days. The LODs were studied for the various elements [19].

Finally, an organic component was evaluated in some samples. Volatile organic compounds (VOCs) were investigated by a purge-and-trap process (with a cryogenic interface) followed by gas chromatography coupled with ion trap mass spectrometry (GC-IT/MS). The sample was purged with helium at a flow rate of up to 40 mL min^−1^ for 10 min while the sample was under agitation. After the purge, desorption occurred: the cryogenic trap, set up at −150 °C, was rapidly heated to 245 °C while backflushing with an inert gas at 4 mL min^−1^ for about 5 min. At the end of the 5 min desorption cycle, the GC temperature program began, and data acquisition started. All the analytical conditions are reported in a previous paper [20]: here, the authors report the main characteristics of the GC-IT/MS system used. A gas chromatograph, Finnigan Trace GC Ultra, equipped with an ion trap mass spectrometry detector, namely, a Polaris Q (Thermo Fisher Scientific, Waltham, MA, USA), a programmed temperature vaporizer (PTV) injector, and dedicated software (Xcalibur 1.4.1, Thermo Fisher, Carlsbad, CA, USA) for data acquisition and analysis, was used. A home column, i.e., fused-silica capillary column with a chemically bonded phase (SE-54, 5% phenyl –95% dimethylpolisiloxane), was used for the analysis [21,22].

## 3. Results and Discussion

### 3.1. Preliminary Study: X-Ray EDS Spectroscopy Analysis

**Figure 2:**
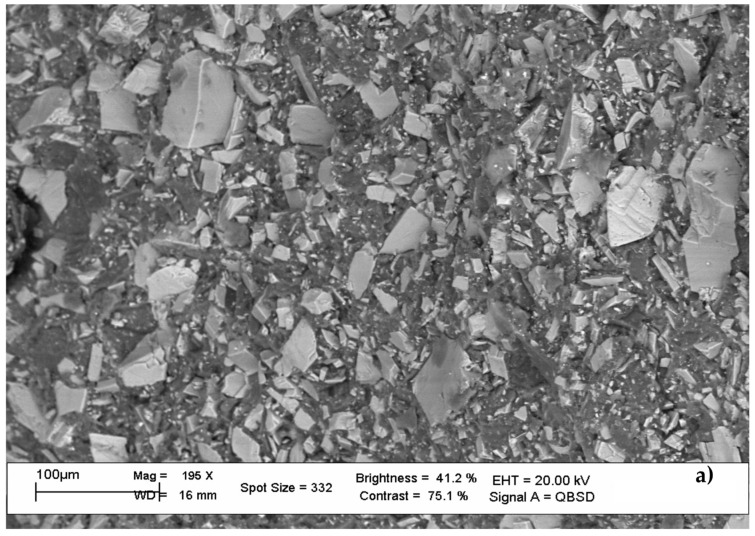

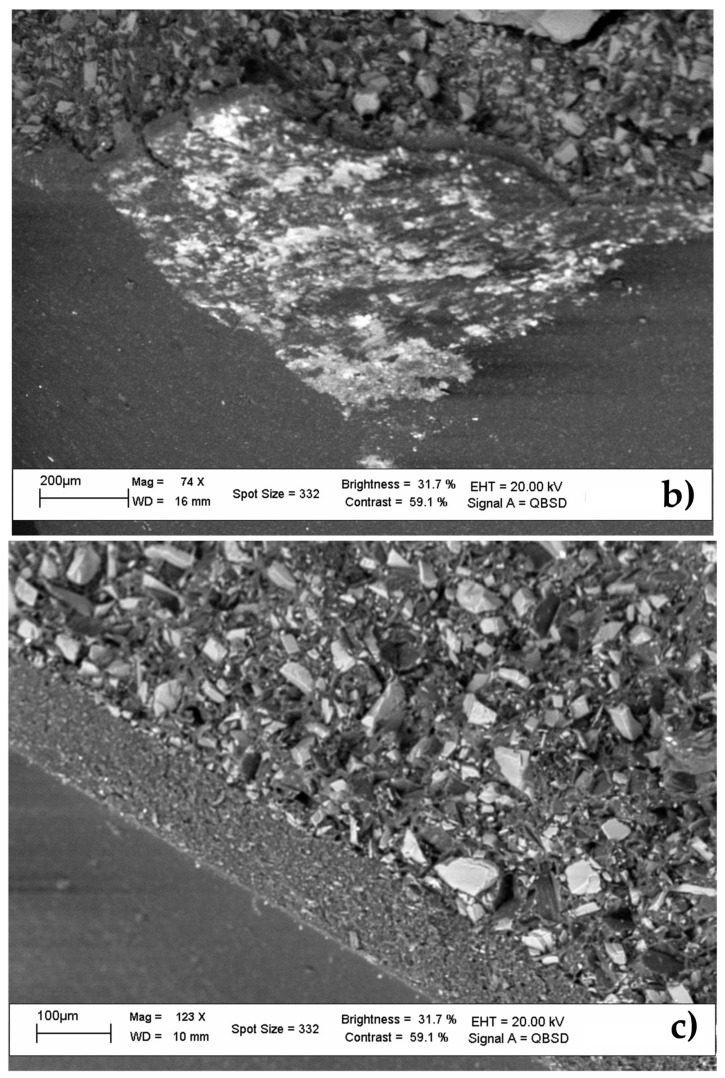

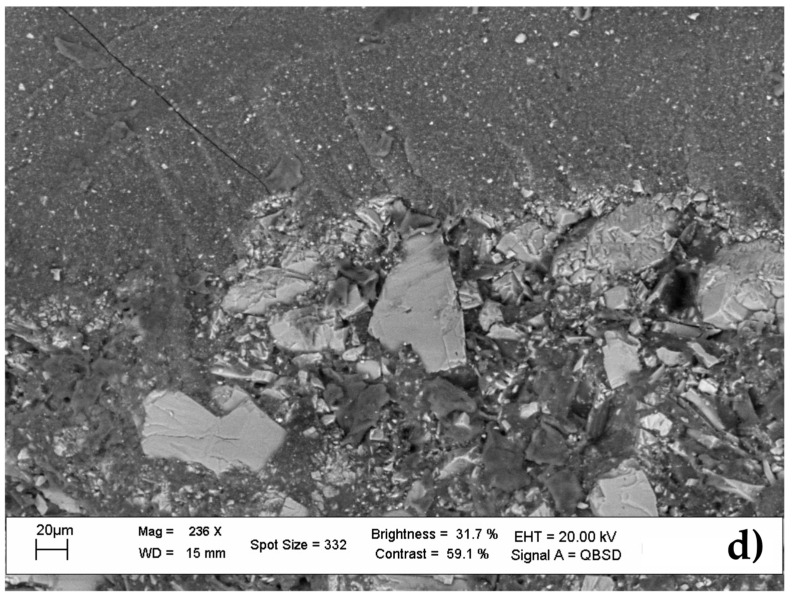
XRF spectra of different sections of a single statuette: (**a**) body, bulk material of mantle; (**b**) body mantle, cross-section, from red varnish to bulk; (**c**) helmet edge, cross-section, brown varnish to bulk; (**d**) neck section, pink varnish to bulk.

First, preliminary experiments using X-rays EDS spectroscopy were performed to test the material. It was not possible to provide any indications on the type of compound (polymer or other) as all the figurines used had different compositions: there was the possibility that the material of which the counterfeit figurines were made came from residues of other types of processing (e.g., industrial discharges), with the presence of toxic metals in its composition. It was therefore decided to use EDS spectroscopy, an analytical method that exploits the X-ray emission generated by an accelerated electron beam incident on the sample. This methodology allowed evaluating the intrinsic structure of various parts of the statuettes as well as the chemical composition (in percentage) of various sections (e.g., various parts of the helmet, internal and external body, cloak, neck of the gladiator statuette). In particular, the composition of the colors that made up a gladiator statue was evaluated. Figure 2 shows the photographs taken under the EDS microscope which highlight and, at the same time, confirm some of the above considerations.

The main composition was mineral, which appeared to be limestone and/or dolomite with different granularities and shapes between the head and the body. The paint covered everything, so it was possible to hypothesize a release from the paints (this could be confirmed by carrying out release tests to assess the danger). Paints were a mixture of dyes and pigments to obtain the color; there was also a more superficial composition composed of metals in micro-powders. Table 1 shows the results found by analyzing the individual parts of a sample, that is, the gladiator: there were very interesting levels of some heavy metals, toxic from a health point of view, such as chromium, lead, cadmium, and arsenic (in trace), as well as some interesting, even if non-toxic, elements (bromine, aluminum, chlorine, iron, zinc). Other very interesting data concern lead (in the golden paint), cadmium (in the red of the coat), and arsenic (in traces) (in the golden paint). Furthermore, chromium, albeit low, was still present in different parts of the figurine as well as in the colored part. Attention to these values depends on the two chemical forms in which chromium is present: Cr^3+^ is non-toxic, while Cr^6+^ is extremely toxic. It is not possible to exclude a priori that part of the chromium found was in the form of Cr^6+^.

### 3.2. Heavy Metal Content and Leaching Tests

For a more systematic approach, the heavy metal level in each sample was first determined. Table 2 shows the inorganic fraction determined in the different statuettes.

First, it could be pointed out that important metals such as Be, Hg, Tl, and V were below their relative LODs, and As, Cd, Mo, Se, and Te were not present in some investigated souvenirs. This preliminary information is important for human health: in fact, even if the polymer that constituted the object was not sufficiently determined, the absence of these species reduces the risk of exposure during handling. More interesting, also for the related implications, were the determinations of organic substances (Table 3): there were quantities lower than those required by the legislation on the safety of toys as well as by the legislation on fabrics [23].

The presence of organic substances of the 1,2-dichloroethane type (species that can cause cancer; easily flammable; harmful even if swallowed; irritating to the eyes, respiratory tract, and skin), xylenes (inflammable species; harmful by inhalation and skin contact; skin irritant), naphthalene (species that is harmful by inhalation and ingestion), phenol (species that is toxic when in contact with the skin and if swallowed; causes burns), and ethylbenzene (species that is harmful by inhalation; easily flammable), in all the samples, poses a major problem regarding the labeling and packaging for the marketing of these products. These interesting results were deepened with a specific study concerning the release of these elements. We must, in fact, consider the playful aspect of the figurines which, once purchased, would be “handled” by both adults and children, above all.

The latter subpopulation is the one most exposed to the release of toxic substances. Therefore, metal release tests were carried out according to international guidelines to evaluate the effective transmission of toxic substances from the colors with which the figurines were painted to the person. Table 4 shows the results obtained for the 24 h, 7-day, and 15-day release tests.

**Figure 3:**
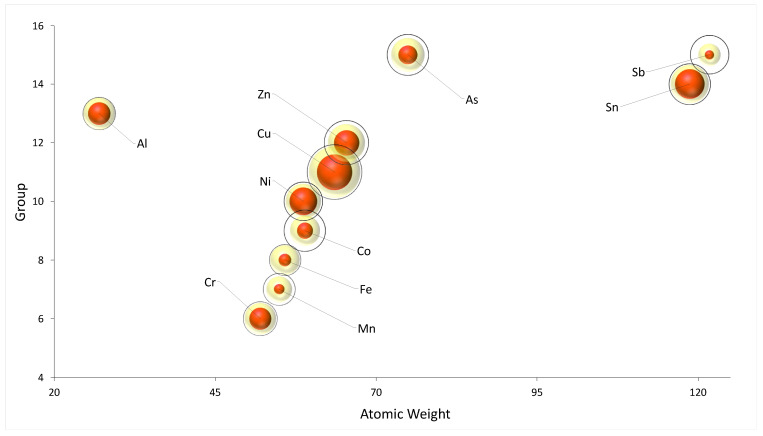
Bubble graph of the leaching tests performed on the different samples. Each bubble is the average of the concentrations determined for each sample in the leaching tests, after normalization. The red bubbles identify the test performed after 24 h, the yellow bubbles after 7 days, and the black circles after 15 days.

Figure 3 shows the normalized representation of the different contributions of each metal during the release tests. Releases at different times are represented with concentric circles (24 h, red bubbles; 7 days, yellow bubbles; 15 days, black circles). As can be seen, Al, Zn, Cu, Ni, Cr, and Sn released most of their concentration within 24 h, whereas Co, Fe, Mn, and Sb tended to be released after several days of use. The latter behavior is quite interesting: Co, Fe, and Mn are in sequence in the periodic table, and Fe and Co are also part of the same group (8B). Finally, only As showed a constant decreasing ratio over the entire period.

The determination of total Cr posed considerations for this element. The chromium value found in a series of samples subjected to transfer tests of variable durations varied between 0.8 and 13.1 μg kg^−1^, whereas in the samples, it varied significantly, between 1.4 and 75 mg kg^−1^. Although the two quantities were very different from each other (about three orders of magnitude), they were still low quantities that did not indicate particular attention from the point of view of the release of toxic substances. Another critical element from the point of view of toxicity was nickel: its values were extremely low in almost all samples, except for sample 278-19 (“centurion, cloak and helmet with red crest-Colosseum base”), which showed a value of 65.3 mg kg^−1^. To obtain a comparison on these data, which is certainly important, the authors took into consideration the legislation on toys (Directive 2009/48/EC of the European Parliament and of the Council of 18 June 2009 on the safety of toys). The reference legislation on toys was used to obtain a comparison of the values of some elements: toys are objects in direct contact with humans, and the transfer of metals is a fundamental parameter. As can be seen from Annex II par. 13, the Ni value was less than the limit value imposed by the standard in any sampling condition of a solid toy. Similar considerations could be extrapolated for other sensitive metals. Another extremely important metal, namely, Pb, showed interesting values, up to 74 mg kg^−1^, especially in the samples of the 278 series; however, this is a value lower than that required by the legislation on toys (always considering the limitation of the type of object), which is 160 mg kg^−1^.

### 3.3. Chemometric Approach

For a better understanding of the leaching surveys, a chemometric approach based on cluster analysis and principal component analysis [24] was tested using Past software (version 4.05, Oslo, Norway) [25]. This methodology was used to assess similarities among different samples as well as what element could be considered fundamental in the release tests.

**Figure 4:**
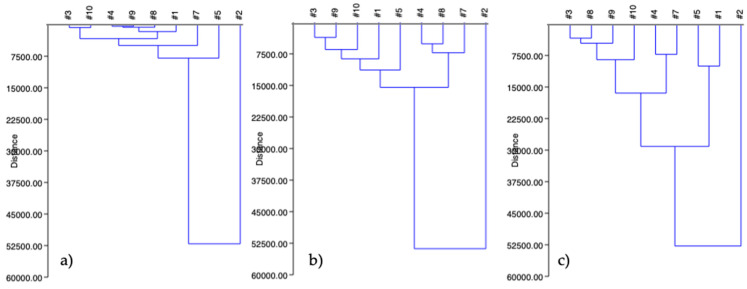
Cluster analysis related to the different samples (**a**) test at 24 h; (**b**) test at 7 days; (**c**) test at 15 days). Samples: #1—Pope with red dress; #2—Pope with white dress; #3—gladiator with black crest; #4—gladiator with red crest; #5—gladiator with laurel crown; #7—centurion; #8—footballer; #9—gladiator with gladius (i.e., a Roman sword); #10—Pope with red cross and zucchetto.

Figure 4 shows the cluster analysis that highlights the presence of three clusters during the tests (Figure 4), whereas Table 5 evidences how these three clusters were grouped (for example, after 24 h of the leaching test, cluster #2 was formed by samples #1, #3, #4, #7, #8, and #9).

Basically, the clusters were made up of the same samples during the three tests, except for sample #1 (from groups #2 to #3): this means that leaching did not affect the main composition profile.

**Figure 5:**
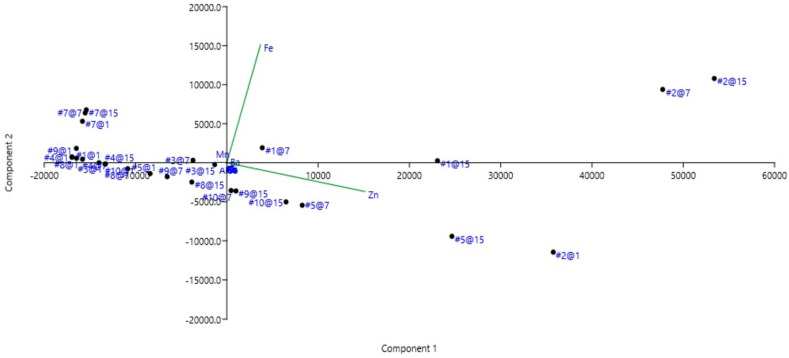
Principal component analysis of the overall data. Each point represents the item (#) and the leaching time (@): for instance, #5@15 means item 5 (gladiator with laurel crown) and leaching time 15 days. Component 1: Zn; Component 2: Fe.

Finally, the authors applied principal component analysis: this highlighted the importance of Zn (component 1) and Fe (component 2). These two components explain 99% of the data, highlighting which elements were important in the leaching tests. Figure 5 shows that cluster #1, formed by sample #2, was sufficiently identified.

## 4. Conclusions

This study found a minimum release of some heavy metals, namely, Cr, Cu, Ni, and Pb, in leaching tests using artificial sweat. These objects are not considered toys but as souvenirs, meaning no legislation prohibits their sale in Italy (and around the world). The authors would like to underline that this study, which is the first in the literature to investigate these statuettes, should be considered preliminary in terms of the analytical implications reported: for instance, the low number of items analyzed (i.e., eight) may not be sufficient to describe the entire situation. Simultaneously, the large variability found for some elements (the main example is V) could be a limitation in this study, and it needs to be investigated in more depth at a later time. The authors wish to emphasize that the main message of this manuscript is the importance of carrying out studies such as this: objects not considered toys but also handled by small children could have possible health consequences in terms of release of (dangerous) chemical compounds. Even if the possibility of carrying these objects in the mouth is only a conjecture (in fact, these objects are not designed to be inserted into the mouths), it should be considered that exposure to hazardous heavy metals (where present) may occur via oral pathways. As these items are considered souvenirs, they are not subjected to any restrictive regulations, as is the case with toys, but (again) they can be manipulated by children. The authors wish to encourage legislators to take action to protect against the presence of chemicals in common consumer goods for the protection of public health. The presence of noxious, toxic, cancer-causing, flammable substances, even if in minimal quantities, must be reported, and the consumer must be informed. The authors wish to recall that the legislation requires the presence of hazardous substances (i.e., harmful, toxic, carcinogenic, flammable) to be indicated, even if in minimal quantities, especially if children handle such objects.

## Figures and Tables

**Table 1:** Levels (as %) of heavy metals determined by X-ray EDS in different parts of a sample, specifically a gladiator.

Section	C	O	Na	Mg	Al	Si	P	S	Cl	K	Ca	Ti	Cr	Fe	Co	Zn	As	Br	Cd	Pb
*Head*																				
black #1	64.5	29.7		1.4	0.2	0.3			0.1	0.2	2.9	0.2		0.4						
black #2	58.9	36.9		0.7		0.7					2.7		0.2							
black #3	57.2	36.8		0.5	0.3	0.7					4.4									
*Crest*																				
black #1	30.6	44.6		0.2		0.6			0.2	0.1	0.7			22.4	0.2			0.4		
black #2	30.2	49.5		8.3		0.2					11.9									
black #3	33.3	46.6				0.8					0.7			18.6						
black #4	45.6	37.8		0.4	0.3	0.7			0.2		1.9	0.3		12.8						
black #5	54.5	39.2		0.7	0.2	0.6					2.0			2.8						
black #6	44.3	42.1		0.5		1.1			0.5		1.2			10.4						
black #7	52.9	39.8	0.2	0.9		0.8		0.2	0.2	0.2	2.3	0.4		2.0						
black #8	50.0	41.9		0.9		0.8		0.2			4.4	0.4		1.4						
*Body*																				
brown #1	55.8	36.4		0.8	0.2	1.3			0.7		4.1	0.6	0.1	0.1						
brown #2	59.3	34.8		0.4		0.8		0.2	0.9		3.2	0.4								
brown #3	28.0	50.5		0.7							20.8									
brown #4	56.2	28.4		0.7		1.1		0.2	0.8		6.1			5.6		1.0				
*Mantle*																				
red #1	46.9	43.2		3.7							5.9							0.3		
red #2	22.3	59.4		1.7							16.6									
red #3	29.3	54.2	0.6	7.6					0.3	0.2	7.8									
red #4	68.1	29.2		1.2							1.7									
red #5	28.5	47.1		6.0					1.9		16.5									
red #6	41.9	30.6		2.5	1.8	5.9			0.6	0.7	7.8	0.8		7.4						
red #7	50.9	42.7		0.8	0.4	1.0		0.2	0.1		2.6	1.2								
red #8	45.6	40.2		1.1	0.4	1.3		0.4	1.1		3.2	0.7		0.5					5.5	
brown #1	54.7	38.6		1.8		2.4			0.3		1.7	0.3		0.2						
brown #2	54.0	37.0		2.5					1.3		5.2									
*Gladius*																				
silver #1	34.1	40.1	1.3	1.7	0.9	0.5	0.7	0.4			7.6	12.9								
silver #2	57.7	36.2		0.3	0.2	0.2		0.3	0.1	0.1	1.9	2.9								
silver #3	44.1	42.7		4.6		0.2		0.1	0.2	0.2	7.7	0.2								
gold #1	59.3	32.3			5.2	0.2			0.2	0.1	0.3	0.2	0.3				0.1			1.9
gold #2	43.2	34.9			1.8						4.3	15.8								
gold #3	49.1	34.9		2.6	0.8	0.9		0.3	0.6	0.4	9.9	0.6								

**Table 2:** Content of heavy metals (mg kg^−1^) determined by ICP-MS in the different samples investigated. Samples: #1—Pope with red dress; #2—Pope with white dress; #3—gladiator with black crest; #4—gladiator with red crest; #5—gladiator with laurel crown; #6—Santa Klaus; #7—centurion; #8—footballer.

#	Al	As	Ba	Be	B	Ca	Cd	Co	Cr	Cu	Fe	Hg	K	Mg	Mn	Mo	Na	Ni	Pb	Sb	Se	Si	Sn	Tl	Te	Ti	V	Zn
#1	360	<0.50	2520	<0.50	2.4	141,750	0.19	15.4	12.2	12	680	<0.10	215	76,300	49.4	<0.50	103	3.1	2.3	9.3	0.98	150	2.8	<0.50	1.62	35.4	<0.50	115
#2	2120	0.66	10,170	<0.50	3.7	86,550	0.11	12.9	46.2	810	780	<0.10	350	46,250	33.9	0.85	280	6.0	3.6	5.6	<0.50	245	12.7	<0.50	1.63	180.0	<0.50	610
#3	690	1.00	140	<0.50	1.6	128,000	0.21	21.9	49.7	37	2480	<0.10	345	62,000	50.4	<0.50	066	4.7	74.1	7.0	<0.50	120	6.2	<0.50	0.62	27.0	<0.50	53
#4	325	0.53	120	<0.50	2.1	122,750	0.15	32.7	15.6	9	580	<0.10	440	59,800	36.5	<0.50	61	3.6	22.8	11.2	<0.50	260	6.2	<0.50	1.41	26.0	<0.50	160
#5	230	<0.50	140	<0.50	2.3	152,350	<0.10	26.6	12.8	10,200	830	<0.10	455	52,800	33.0	0.57	110	4.8	11.7	7.0	<0.50	275	8.3	<0.50	<0.50	68.5	<0.50	1100
#6	200	1.90	44	<0.50	3.3	224,000	0.21	16.9	1.4	125	510	<0.10	265	7400	145.0	0.61	43	4.2	2.9	1.0	0.71	235	2.8	<0.50	<0.50	13.2	<0.50	30
#7	540	0.58	42	<0.50	2.4	175,500	<0.10	17.0	75.0	3700	1240	<0.10	395	3930	22.7	8.30	220	65.3	61.8	3.5	0.58	440	26.3	<0.50	0.75	17.5	<0.50	29
#8	385	<0.50	235	<0.50	2.8	178,000	0.20	14.2	4.9	54	655	<0.10	320	25,800	42.7	<0.50	185	5.0	21.4	12.4	0.52	150	46.0	<0.50	<0.50	18.7	<0.50	140
*x^1^*	*606*	*0.90*	*1676*		*2.6*	*151,113*	*0.20*	*19.7*	*27.2*	*1868*	*969*		*348*	*41,785*	*51.7*	*2.60*	*134*	*12.1*	*25.1*	*7.1*	*0.70*	*234*	*13.9*		*1.21*	*48.3*		*280*
*s.d.^2^*	*632*	*0.57*	*3534*		*0.67*	*41,793*	*0.04*	*6.9*	*26.4*	*3595*	*650*		*82*	*26,556*	*38.8*	*3.81*	*85*	*21.5*	*27.8*	*3.82*	*0.20*	*101*	*15.0*		*0.49*	*56*		*382*
*min^3^*	*200*	*0.53*	*42*		*1.6*	*86,550*	*0.11*	*12.9*	*1.4*	*9*	*510*		*215*	*3930*	*22.7*	*0.57*	*43*	*3.1*	*2.3*	*1.0*	*0.52*	*120*	*2.8*		*0.62*	*13.2*		*29*
*max^4^*	*2120*	*1.9*	*10,170*		*3.7*	*224,000*	*0.21*	*32.7*	*75*	*10,200*	*2480*		*455*	*76,300*	*145*	*8.30*	*280*	*65.3*	*74.1*	*12.4*	*0.98*	*440*	*46.0*		*1.63*	*180*		*1100*
*cv^5^*	*104.3*	*61.1*	*210.8*		*26.1*	*27.7*	*22.5*	*34.9*	*97.0*	*192.4*	*67.0*		*23.7*	*63.6*	*75.0*	*147.7*	*64.0*	*178.0*	*111.0*	*53.6*	*29.3*	*43.3*	*108.1*		*40.3*	*115.9*		*136.5*

^1^ x: mean value; ^2^ min: minimum value; ^3^ max: maximum value; ^4^ s.d.: standard deviation; ^5^ cv%: coefficient of variation.

**Table 3:** Content of organic compounds (µg kg^−1^; n.d., not detected) determined by means of GC-IT/MS in the different samples investigated. For sample identification, see Table 2.

#	CH_2_ClCH_2_Cl	CHCl_3_	Ethylbenzene	Toluene	Xylenes	Styrene	Naphthalene	Phenanthrene	Pyrene	Fluoranthene	Chrysene	Phenol	2-Methylphenol
#1	10,395	0.81	8.1	1.3	8.3	2.3	3470	59	n.d.	n.d.	n.d.	1425	n.d.
#2	11,055	0.86	5.0	1.5	5.1	2.3	2260	99	n.d.	255	n.d.	1550	n.d.
#3	14,920	n.d.	7.8	1.4	9.8	2.3	3240	6570	n.d.	n.d.	180	3200	n.d.
#4	6185	0.41	3.5	0.92	3.9	8.6	3675	n.d.	n.d.	n.d.	155	2260	n.d.
#5	n.d.	0.81	n.d.	n.d.	n.d.	n.d.	3285	n.d.	n.d.	n.d.	397	3225	n.d.
#6	n.d.	0.86	n.d.	n.d.	n.d.	n.d.	1585	n.d.	n.d.	n.d.	n.d.	5010	1465
#7	n.d.	n.d.	n.d.	n.d.	n.d.	n.d.	662	328	235	n.d.	n.d.	5645	n.d.
#8	n.d.	0.41	n.d.	n.d.	n.d.	n.d.	1285	n.d.	n.d.	n.d.	n.d.	6265	n.d.
*x^1^*	*10,639*	*0.69*	*6.1*	*1.3*	*6.8*	*3.9*	*2433*	*1764*	*235*	*255*	*244*	*3573*	*1465*
*s.d.^2^*	*3574*	*0.22*	*2.2*	*0.25*	*2.7*	*3.2*	*831*	*3207*			*133*	*1861*	
*min^3^*	*6185*	*0.41*	*3.5*	*0.92*	*3.9*	*2.3*	*662*	*59*			*155*	*1425*	
*max^4^*	*14,920*	*0.86*	*8.1*	*1.5*	*9.8*	*8.6*	*3675*	*6570*			*397*	*6265*	
*cv^5^*	*33.6*	*31.8*	*36.5*	*19.8*	*40.5*	*81.3*	*47.1*	*181.8*			*54.5*	*52.1*	

**Table 4:** Heavy metal levels (µg kg^−1^) determined by ICP-MS after leaching tests at different times on the investigated samples, performed according to the CNR IRSA method [15]. For sample identification, see Table 2. Release tests were not performed on sample #6, i.e., Sant Klaus, due to the sample availability; #9—gladiator with *gladius* (i.e., a Roman sword); and #10—Pope with red cross and zucchetto.

**#**	**Ag**			**Al**			**As**			**Ba**			**Be**			**Cd**			**Co**		
	24 h	7 days	15 days	24 h	7 days	15 days	24 h	7 days	15 days	24 h	7 days	15 days	24 h	7 days	15 days	24 h	7 days	15 days	24 h	7 days	15 days
#1	< 0.5	< 0.5	< 0.5	9.2	81.5	81.9	< 0.5	0.52	0.64	1145	2105	2185	< 0.5	< 0.5	< 0.5	< 0.3	< 0.3	< 0.3	1.7	10.1	25.6
#2	< 0.5	< 0.5	< 0.5	47.1	91.5	120.0	< 0.5	1.6	1.6	8.5	18.9	20.4	< 0.5	< 0.5	< 0.5	< 0.3	< 0.3	< 0.3	5.6	23.7	56.0
#3	< 0.5	< 0.5	< 0.5	32.8	115.0	120.1	< 0.5	0.69	1.1	5.6	8.6	9.9	< 0.5	< 0.5	< 0.5	< 0.3	< 0.3	< 0.3	2.4	15.1	28.5
#4	< 0.5	< 0.5	< 0.5	26.5	125.0	155.2	< 0.5	1.4	1.5	23.1	42.8	48.4	< 0.5	< 0.5	< 0.5	< 0.3	< 0.3	< 0.3	6.2	29.8	61.9
#5	< 0.5	< 0.5	< 0.5	39.6	41.9	64.1	< 0.5	1.4	1.3	18.4	35.0	40.6	< 0.5	< 0.5	< 0.5	< 0.3	< 0.3	< 0.3	26.7	115.4	219.9
#7	< 0.5	< 0.5	< 0.5	27.0	29.1	40.9	< 0.5	6.1	7.6	33.9	46.4	50.6	< 0.5	< 0.5	< 0.5	< 0.3	< 0.3	< 0.3	33.9	72.0	110.3
#8	< 0.5	< 0.5	< 0.5	24.0	39.4	41.7	< 0.5	1.3	1.8	90.2	205.3	260.2	< 0.5	< 0.5	< 0.5	< 0.3	< 0.3	< 0.3	3.0	17.7	40.5
#9	< 0.5	< 0.5	< 0.5	392.3	749.8	770.1	< 0.5	0.87	6.0	54.6	75.3	86.2	< 0.5	< 0.5	< 0.5	< 0.3	< 0.3	< 0.3	45.9	161.9	300.1
#10	< 0.5	< 0.5	< 0.5	53.1	53.5	35.9	< 0.5	0.61	0.82	11.8	19.2	21.2	< 0.5	< 0.5	< 0.5	< 0.3	< 0.3	< 0.3	2.6	17.2	41.7
**#**	**Cr**			**Cu**			**Fe**			**Hg**			**Mn**			**Mo**			**Ni**		
	24 h	7 days	15 days	24 h	7 days	15 days	24 h	7 days	15 days	24 h	7 days	15 days	24 h	7 days	15 days	24 h	7 days	15 days	24 h	7 days	15 days
#1	< 0.5	< 0.5	0.69	1.5	< 1.0	2.6	1235	6130	9060	< 0.5	< 0.5	< 0.5	10.5	80.5	155.0	< 0.5	< 0.5	< 0.5	2.2	5.9	10.1
#2	0.82	1.3	2.0	4.8	7.4	8.9	720.2	23,810	26,500	< 0.5	< 0.5	< 0.5	7.8	440.2	760.3	< 0.5	< 0.5	< 0.5	1.9	8.5	10.4
#3	< 0.5	< 0.5	< 0.5	7.8	4.7	6.0	46.6	2795	2830	< 0.5	< 0.5	< 0.5	6.0	32.4	43.6	< 0.5	< 0.5	< 0.5	1.3	2.3	2.6
#4	0.73	2.5	5.0	8.4	11.3	11.8	28.2	59.1	65.3	< 0.5	< 0.5	< 0.5	4.8	15.8	18.0	< 0.5	< 0.5	< 0.5	25.3	33.8	40.5
#5	0.55	1.5	1.8	17.1	18.0	18.4	24.0	26.7	51.2	< 0.5	< 0.5	< 0.5	6.0	12.0	15.2	< 0.5	< 0.5	< 0.5	1.8	2.0	3.8
#7	< 0.5	< 0.5	< 0.5	7.6	8.3	9.0	4780	5890	6300	< 0.5	< 0.5	< 0.5	65.8	230.1	275.4	< 0.5	< 0.5	< 0.5	3.0	3.8	4.3
#8	4.7	12.6	13.1	16.0	16.6	22.3	22.8	47.4	48.5	< 0.5	< 0.5	< 0.5	8.6	18.8	24.6	< 0.5	< 0.5	< 0.5	8.6	16.0	20.6
#9	1.7	1.7	2.1	31.1	31.7	34.9	79.1	83.0	90.1	< 0.5	< 0.5	< 0.5	15.3	19.1	20.7	< 0.5	< 0.5	< 0.5	6.6	9.8	10.9
#10	< 0.5	0.82	0.90	6.9	< 1.0	< 1.0	32.7	18.8	25.7	< 0.5	< 0.5	< 0.5	4.5	7.8	7.8	< 0.5	< 0.5	< 0.5	0.94	1.5	1.6
**#**	**Pb**			**Sb**			**Sn**			**Te**			**Ti**			**V**			**Zn**		
	24 h	7 days	15 days	24 h	7 days	15 days	24 h	7 days	15 days	24 h	7 days	15 days	24 h	7 days	15 days	24 h	7 days	15 days	24 h	7 days	15 days
#1	< 0.5	0.54	0.50	1.5	13.3	44.8	0.5	1.5	2.6	< 0.5	< 0.5	< 0.5	< 0.3	< 0.3	< 0.3	1.8	2.8	3.7	280.2	20,045	39,085
#2	0.8	8.2	11.5	< 0.5	1.6	1.6	< 0.5	3.0	3.6	< 0.5	< 0.5	< 0.5	< 0.3	< 0.3	< 0.3	1.8	2.7	3.1	54,185	60,890	66,050
#3	1.5	1.9	2.2	< 0.5	< 0.5	< 0.5	0.73	0.82	0.84	< 0.5	< 0.5	< 0.5	< 0.3	< 0.3	< 0.3	2.1	5.2	5.6	3130	13,060	15,500
#4	1.5	2.2	2.6	2.4	24.2	75.5	4.2	8.4	10.7	< 0.5	< 0.5	< 0.5	< 0.3	< 0.3	< 0.3	1.8	7.3	7.9	86.7	1270	3850
#5	2.7	3.0	3.2	< 0.5	< 0.5	0.73	0.92	1.0	1.3	< 0.5	< 0.5	< 0.5	< 0.3	< 0.3	< 0.3	1.9	6.5	6.9	8900	26,045	42,920
#7	13.8	18.0	22.2	2.4	6.1	13.8	4.2	5.2	5.7	< 0.5	< 0.5	< 0.5	< 0.3	< 0.3	< 0.3	4.8	4.8	4.9	112.4	145.3	173.2
#8	20.6	44.0	47.4	1.8	16.6	56.0	1.8	3.2	3.7	< 0.5	< 0.5	< 0.5	< 0.3	< 0.3	< 0.3	2.9	8.0	9.7	605	6370	13,590
#9	8.7	10.9	12.6	0.66	0.93	1.4	2.5	2.5	2.1	< 0.5	< 0.5	< 0.5	< 0.3	< 0.3	< 0.3	4.5	4.9	5.5	65.5	10,810	18,560
#10	1.6	2.0	2.5	< 0.5	< 0.5	0.52	< 0.5	0.53	0.54	< 0.5	< 0.5	< 0.5	< 0.3	< 0.3	< 0.3	1.7	3.5	4.5	3820	18,030	24,220

**Table 5:** Representation of the cluster analysis during the three leaching tests. The different colors represent the three clusters. For sample identification, see Figure 4.

Sample	24 h	7 Days	15 Days
#1	cluster 2	cluster 3	cluster 3
#2	cluster 1	cluster 1	cluster 1
#3	cluster 2	cluster 3	cluster 2
#4	cluster 2	cluster 2	cluster 2
#5	cluster 3	cluster 3	cluster 3
#7	cluster 2	cluster 2	cluster 2
#8	cluster 2	cluster 2	cluster 2
#9	cluster 2	cluster 2	cluster 2
#10	cluster 3	cluster 3	cluster 3

The colors mean the different groups.

## Data Availability

All the data are reported in the paper.
